# Selfish genetic elements

**DOI:** 10.1371/journal.pgen.1007700

**Published:** 2018-11-15

**Authors:** J. Arvid Ågren, Andrew G. Clark

**Affiliations:** Department of Molecular Biology and Genetics, Cornell University, Ithaca, NY

## Abstract

**Selfish genetic elements** (historically also referred to as selfish genes, ultra-selfish genes, selfish DNA, parasitic DNA, genomic outlaws) are genetic segments that can enhance their own transmission at the expense of other genes in the genome, even if this has no or a negative effect on organismal fitness. [1–6] Genomes have traditionally been viewed as cohesive units, with genes acting together to improve the fitness of the organism. However, when genes have some control over their own transmission, the rules can change, and so just like all social groups, genomes are vulnerable to selfish behaviour by their parts. Early observations of selfish genetic elements were made almost a century ago, but the topic did not get widespread attention until several decades later. Inspired by the gene-centred views of evolution popularized by George Williams[7] and Richard Dawkins,[8] two papers were published back-to-back in *Nature* in 1980—by Leslie Orgel and Francis Crick[9] and Ford Doolittle and Carmen Sapienza[10] respectively—introducing the concept of selfish genetic elements (at the time called “selfish DNA”) to the wider scientific community. Both papers emphasized that genes can spread in a population regardless of their effect on organismal fitness as long as they have a transmission advantage. Selfish genetic elements have now been described in most groups of organisms, and they demonstrate a remarkable diversity in the ways by which they promote their own transmission.[11] Though long dismissed as genetic curiosities, with little relevance for evolution, they are now recognized to affect a wide swath of biological processes, ranging from genome size and architecture to speciation.[12]

This is a Topic Page article for *PLOS Genetics*.

## History

### Early observations

Observations of what we now refer to as selfish genetic elements go back to the early days in the history of genetics. Already in 1928, Russian geneticist Sergey Gershenson reported the discovery of a driving X chromosome in *Drosophila obscura*.[13] Crucially, he noted that the resulting female-biased sex ratio may drive a population extinct (see Consequences to the Host of Selfish Genetic Elements: Species extinction below). The earliest clear statement of how chromosomes may spread in a population not because of their positive fitness effects on the individual organism, but because of their own”parasitic” nature came from the Swedish botanist and cytogeneticist Gunnar Östergren in 1945.[14] Discussing B chromosomes in plants he wrote:

”In many cases these chromosomes have no useful function at all to the species carrying them, but that they often lead an exclusively parasitic existence … [B chromosomes] need not be useful for the plants. They need only be useful to themselves.”—Gunnar Östergren[14]

Around the same time, several other examples of selfish genetic elements were reported. For example, the American maize geneticist Marcus Rhoades described how chromosomal knobs led to female meiotic drive in maize.[15] Similarly, this was also when it was first suggested that a conflict between uniparentally inherited mitochondrial genes and biparentally inherited nuclear genes could lead to cytoplasmic male sterility in plants.[16] Then, in the early 1950s, Barbara McClintock published a series of papers describing the existence of transposable elements, which are now recognized to be among the most successful selfish genetic elements. [17,18] The discovery of transposable elements led to her being awarded the Nobel Prize in Medicine or Physiology in 1983.

### Conceptual developments

The empirical study of selfish genetic elements benefited greatly from the emergence of the so-called gene’s-eye view of evolution in the nineteen sixties and seventies.[19] In contrast with Darwin’s original formulation of the theory of evolution by natural selection that focused on individual organisms, the gene’s-eye view takes the gene to be the central unit of selection in evolution.[20] It conceives evolution by natural selection as a process involving two separate entities: replicators (entities that produce faithful copies of themselves, usually genes) and vehicles (or interactors; entities that interact with the ecological environment, usually organisms).[21–23] Since organisms are temporary occurrences, present in one generation and gone in the next, genes (replicators) are the only entity faithfully transmitted from parent to offspring. Viewing evolution as a struggle between competing replicators made it easier to recognize that not all genes in an organism would share the same evolutionary fate.

**Fig 1:**
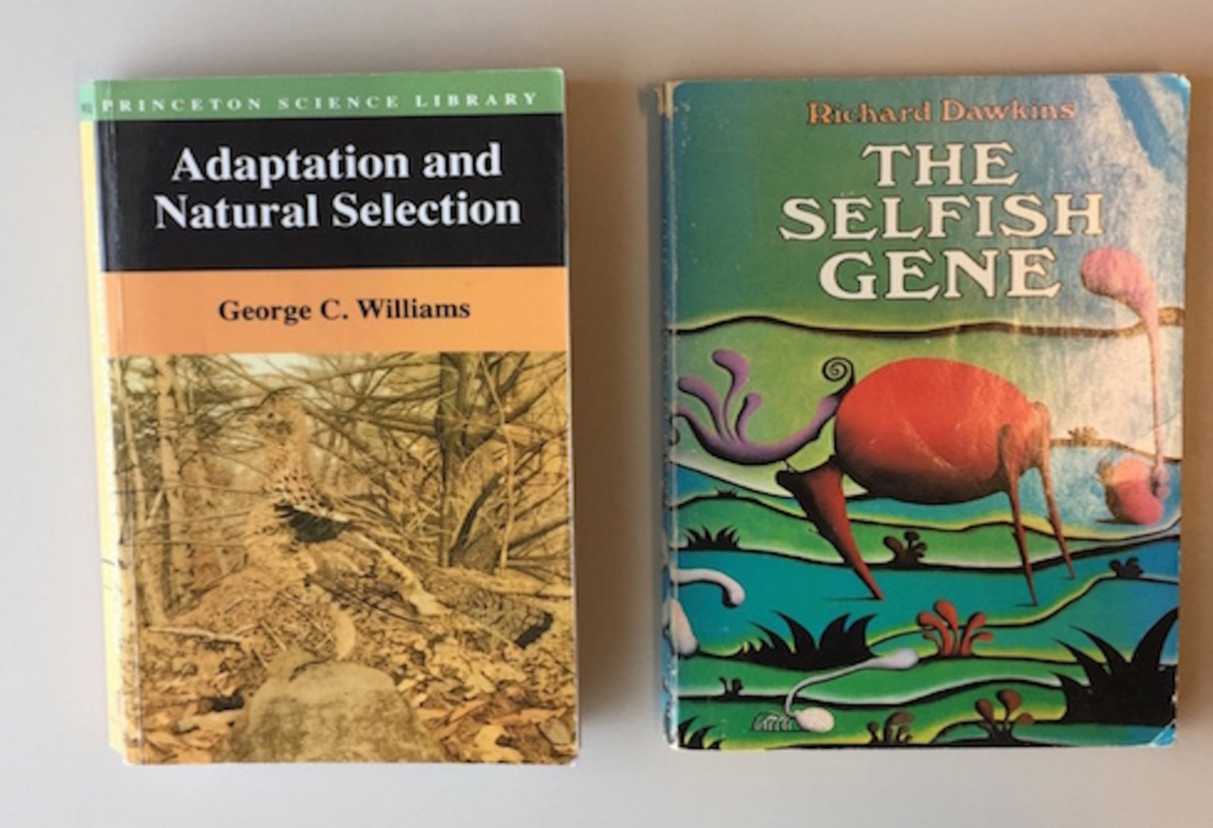
George Williams' *Adaptation and Natural Selection* (1966) and Richard Dawkins' *The Selfish Gene* (1976) were instrumental in introducing the gene's-eye view to evolutionary biology.

The gene’s-eye view was a synthesis of the population genetic models of the modern synthesis, in particular the work of RA Fisher, and the social evolution models of Bill Hamilton. The view was popularized by George Williams' *Adaptation and Natural Selection*[7] and Richard Dawkins‘ best seller *The Selfish Gene* (Fig 1).[8] Dawkins summarized a key benefit from the gene’s-eye view as follows:

”If we allow ourselves the license of talking about genes as if they had conscious aims, always reassuring ourselves that we could translate our sloppy language back into respectable terms if we wanted to, we can ask the question, what is a single selfish gene trying to do?”—Richard Dawkins *The Selfish Gene* [8] p. 88

In 1980, two high profile papers published back-to-back in *Nature* by Leslie Orgel and Francis Crick, and Ford Doolittle and Carmen Sapienza respectively, brought the study of selfish genetic elements to the centre of biological debate.[9–10] The papers took their starting point in the contemporary debate of the so-called C-value paradox (see below), the lack of correlation between genome size and perceived complexity of a species. Both papers attempted to counter the prevailing view of the time that the presence of differential amounts of non-coding DNA and transposable elements is best explained from the perspective of individual fitness, described as the”phenotypic paradigm” by Doolittle and Sapienza. Instead, the authors argued that much of the genetic material in eukaryotic genomes persists, not because of its phenotypic effects, but can be understood from a gene’s-eye view, without invoking individual-level explanations. The two papers led to a series of exchanges in *Nature*.[24–27]

### Current views

If the Selfish DNA papers marked the beginning of the serious study of selfish genetic elements, the subsequent decades have seen an explosion in theoretical advances and empirical discoveries. Leda Cosmides and John Tooby wrote a landmark review about the conflict between maternally inherited cytoplasmic genes and biparentally inherited nuclear genes.[28] The paper also provided a comprehensive introduction to the logic of genomic conflicts, foreshadowing many themes that would later be subject of much research. Then in 1988, John H. Werren and colleagues wrote the first major empirical review of the topic.[1] This paper achieved three things. First, it coined the term selfish genetic element, putting an end to a sometimes confusingly diverse terminology (selfish genes, ultra-selfish genes, selfish DNA, parasitic DNA, genomic outlaws). Second, it formally defined the concept of selfish genetic elements. Finally, it was the first paper to bring together all different kinds of selfish genetic elements known at the time (genomic imprinting, for example, was not covered). In the late 1980s, most molecular biologists considered selfish genetic element to be the exception, and that genomes were best thought of as highly integrated networks with a coherent effect on organismal fitness. In 2006, when Austin Burt and Robert Trivers published the first book-length treatment of the topic, a comprehensive piece that remains the go-to source on the topic, the tide was changing. While their role in evolution long remained controversial, in a recent review, a century after their first discovery, William R. Rice concluded that “nothing in genetics makes sense except in the light of genomic conflicts”.[29]

## The logic of selfish genetic elements

Though selfish genetic elements show a remarkable diversity in the way they promote their own transmission, some generalizations about their biology can be made. In a classic 2001 review, Gregory D.D. Hurst and John H. Werren proposed two ‘rules’ of selfish genetic elements.[4]

### Rule 1: The spread of selfish genetic elements requires sex and outbreeding

Sexual reproduction involves the mixing of genes from two individuals. According to Mendel’s Law of Segregation, alleles in a sexually reproducing organism have a 50% chance of being passed from parent to offspring. Meiosis is therefore sometimes referred to as “fair”.[30]

Highly self-fertilizing or asexual genomes are expected to experience less conflict between selfish genetic elements and the rest of the host genome than outcrossing sexual genomes.[31–33] There are several reasons for this. First, sex and outcrossing put selfish genetic elements into new genetic lineages. In contrast, in a highly selfing or asexual lineage, any selfish genetic element is essentially stuck in that lineage, which should increase variation in fitness among individuals. The increased variation should result in stronger purifying selection in selfers/asexuals, as a lineage without the selfish genetic elements should out-compete a lineage with the selfish genetic element. Second, the increased homozygosity in selfers removes the opportunity for competition among homologous alleles. Third, theoretical work has shown that the greater linkage disequilibrium in selfing compared to outcrossing genomes may in some, albeit rather limited, cases cause selection for reduced transposition rates.[34] Overall, this reasoning leads to the prediction that asexuals/selfers should experience a lower load of selfish genetic elements. One caveat to this is that the evolution of selfing is associated with a reduction in the effective population size.[35] A reduction in the effective population size should reduce the efficacy of selection and therefore leads to the opposite prediction: higher accumulation of selfish genetic elements in selfers relative to outcrossers. Empirical evidence for the importance of sex and outcrossing comes from a variety of selfish genetic elements, including transposable elements,[36,37] self-promoting plasmids,[38] and B chromosomes.[39]

### Rule 2: The presence of selfish genetic elements is often revealed in hybrids

The presence of selfish genetic elements can be difficult to detect in natural populations. Instead, their phenotypic consequences often become apparent in hybrids. The first reasons for this is that some selfish genetic elements rapidly sweep to fixation, and the phenotypic effects will therefore not be segregating the in the population. Hybridization events, however, will produce offspring with and without the selfish genetic elements and so reveal their presence. The second reason is that host genomes have evolved mechanisms to suppress the activity of the selfish genetic elements, for example the small RNA administered silencing of transposable elements.[40] The co-evolution between selfish genetic elements and their suppressors can be rapid, and follow a Red Queen dynamics, which may mask the presence of selfish genetic elements in a population. Hybrid offspring, on the other hand, may inherit a given selfish genetic element, but not the corresponding suppressor and so reveal the phenotypic effect of the selfish genetic element.[41,42]

## Examples of selfish genetic elements

### Segregation distorters

**Fig 2:**
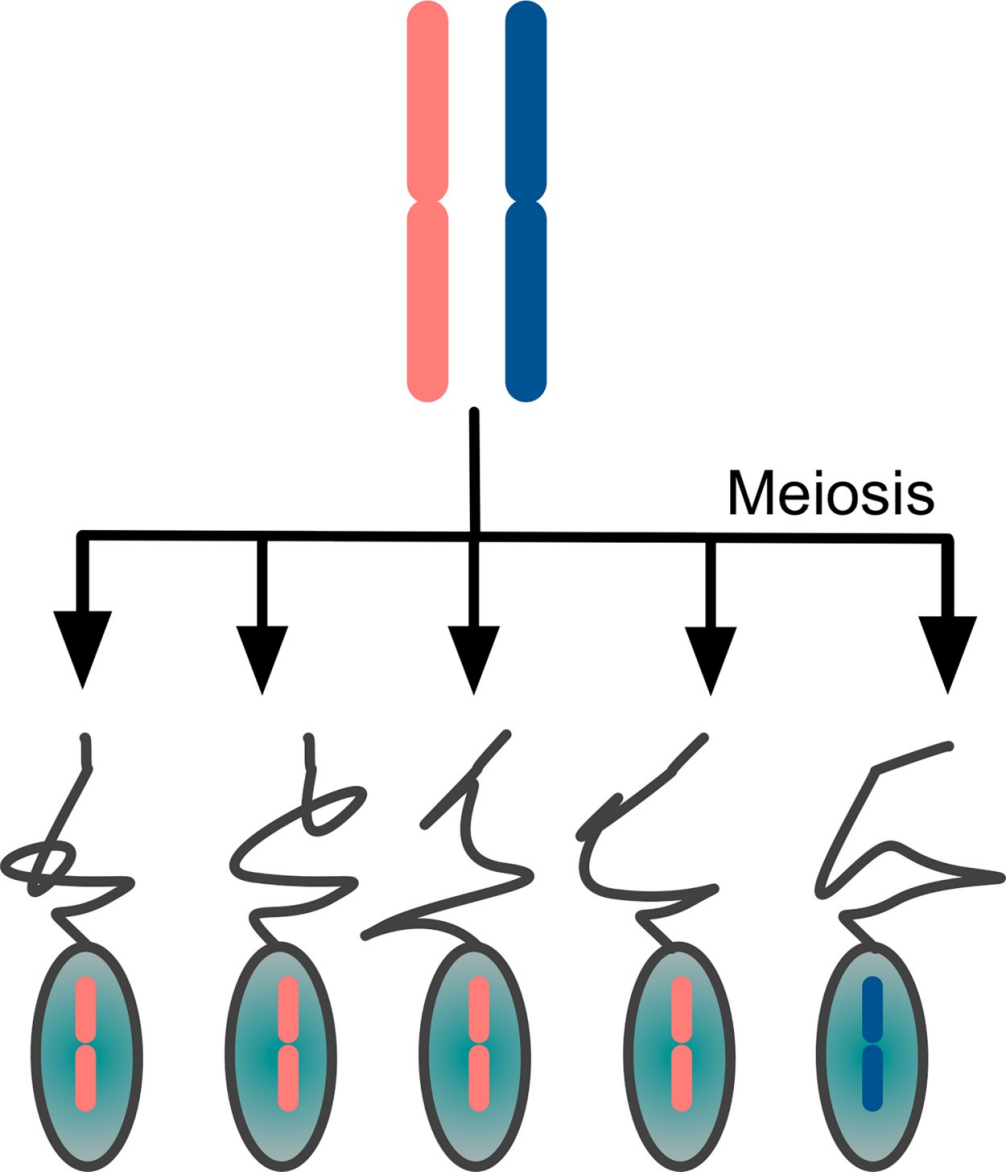
Segregation distorters (here shown in red) get transmitted to >50% of the gametes.

Some selfish genetic elements manipulate the genetic transmission process to their own advantage, and so end up being overrepresented in the gametes (Fig 2). Such distortion can occur in various ways, and the umbrella term that encompasses all of them is segregation distortion. Some elements can preferentially be transmitted in egg cells as opposed to polar bodies during meiosis, where only the former will be fertilized and transmitted to the next generation. Any gene that can manipulate the odds of ending up in the egg rather than the polar body will have a transmission advantage, and will increase in frequency in a population.

Segregation distortion can happen in several ways. When this process occurs during meiosis it is referred to as meiotic drive. Many forms of segregation distortion occur in male gamete formation, where there is differential mortality of spermatids during the process of sperm maturation or spermiogenesis. The Segregation Distorter (SD) in *Drosophila melanogaster* is the best studied example, and it involves a nuclear envelope protein Ran-GAP and the X-linked repeat array called Responder (Rsp), where the SD allele of Ran-GAP favors its own transmission only in the presence of a Rspsensitive allele on the homologous chromosome.[43–48] SD acts to kill RSP^sensitive^ sperm, in a post-meiotic process (hence it is not strictly speaking meiotic drive). Systems like this can have interesting rock-paper-scissors dynamics, oscillating between the SD-RSP^insensitive^, SD+-RSP^insensitive^ and SD+-RSP^sensitive^ haplotypes. The SD-RSP^sensitive^ haplotype is not seen because it essentially commits suicide.

When segregation distortion acts on sex chromosomes, they can skew the sex ratio. The SR system in *Drosophila pseudoobscura*, for example, is on the X chromosome, and X^SR^/Y males produce only daughters, whereas females undergo normal meiosis with Mendelian proportions of gametes.[49,50] Segregation distortion systems would drive the favored allele to fixation, except that most of the cases where these systems have been identified have the driven allele opposed by some other selective force. One example is the lethality of the t-haplotype in mice,[51]another is the effect on male fertility of the Sex Ratio system in *D*. *pseudoobscura*.[49]

### Homing endonucleases

**Fig 3:**
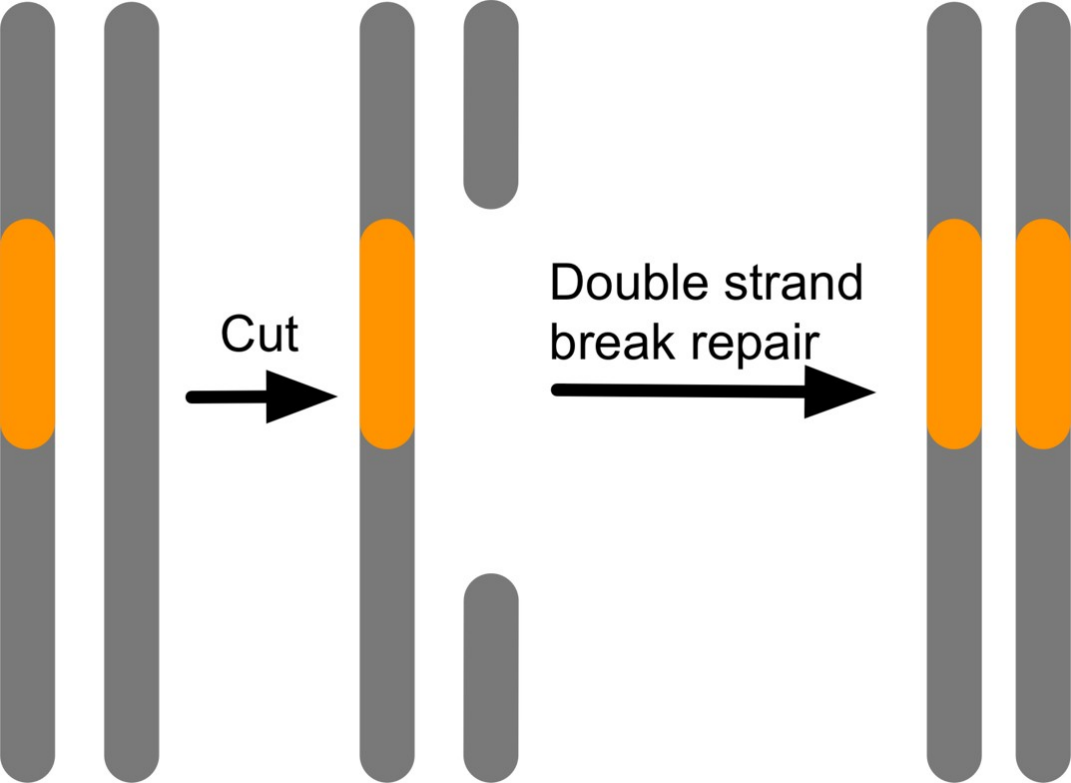
Homing endonucleases can recognize a target sequence, cut it, and then use it own sequence as a template during double strand break repair. This converts a heterozygote into a homozygote.

A phenomenon closely related to segregation distortion is homing endonucleases.[52–54] These are enzymes that cut DNA in a sequence-specific way, and those cuts, generally double-strand breaks, are then “healed” by the regular DNA repair machinery. Homing endonucleases insert themselves into the genome at the site homologous to the first insertion site, resulting in a conversion of a heterozygote into a homozygote bearing a copy of the homing endonuclease on both homologous chromosomes (Fig 3). This gives homing endonucleases an allele frequency dynamics rather similar to a segregation distortion system, and generally unless opposed by strong countervailing selection, they are expected to go to fixation in a population. CRISPR-Cas9 technology allows the artificial construction of homing endonuclease systems. These so-called “gene drive” systems pose a combination of great promise for biocontrol but also potential risk[55,56] (see below).

### Transposable elements

**Fig 4:**
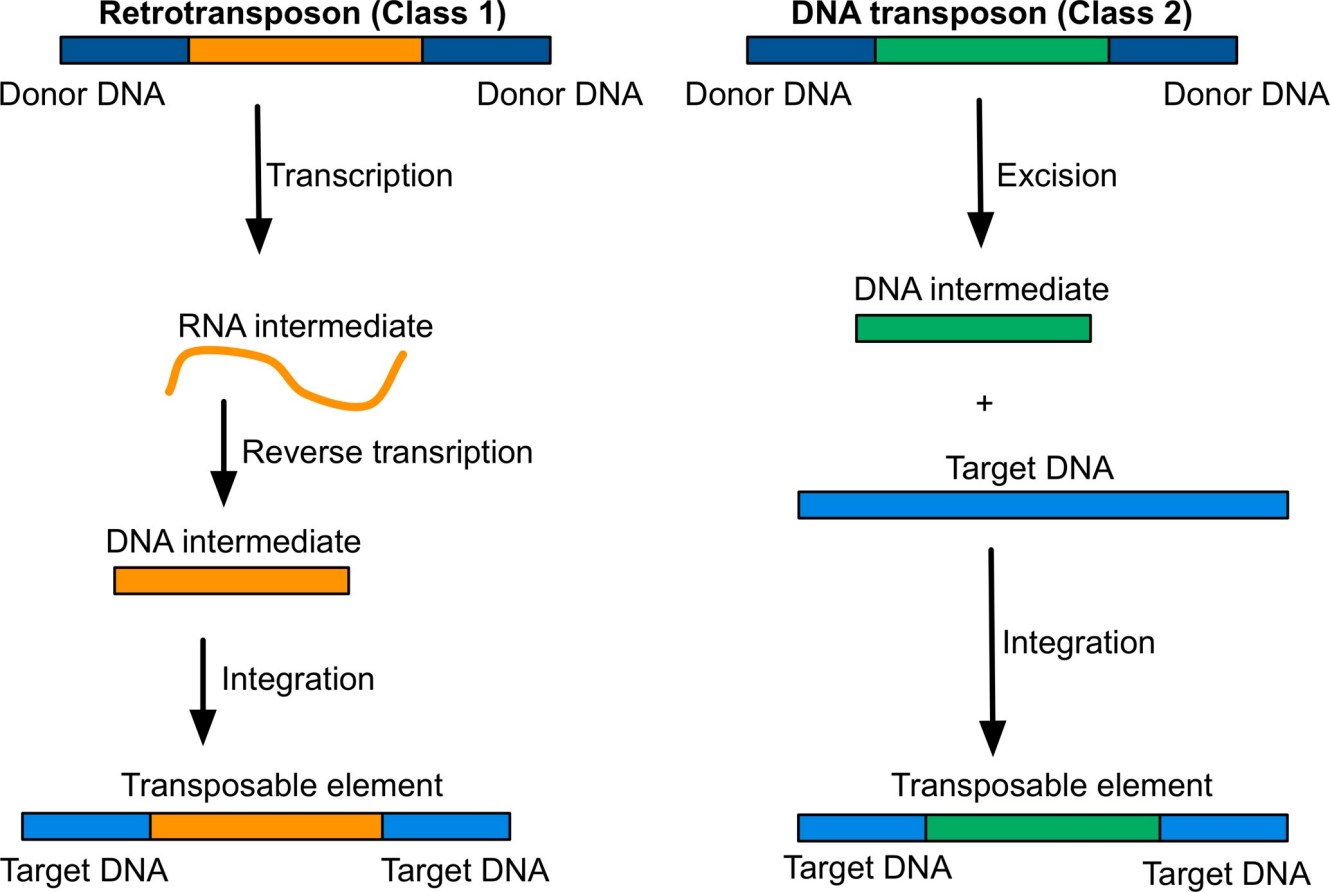
Transposable elements self-replicate through two main mechanisms: via an RNA intermediate ("copy-and-paste"; class 1) or straight excision-insertion ("cut-and-paste"; class 2).

Transposable elements (TEs) include a wide variety of DNA sequences that all have the ability to move to new locations in the genome of their host. Transposons do this by a direct cut-and-paste mechanism, whereas retrotransposons need to produce an RNA intermediate to move. TEs were first discovered in maize by Barbara McClintock in the 1940s[17] and their ability to occur in both active and quiescent states in the genome was also first elucidated by McClintock.[57] TEs have been referred to as selfish genetic elements because they have some control over their own propagation in the genome (Fig 4). Most random insertions into the genome appear to be relatively innocuous, but they can disrupt critical gene functions with devastating results.[58] For example, TEs have been linked to a variety of human diseases, ranging from cancer to haemophilia.[59] TEs that tend to avoid disrupting vital functions in the genome tend to remain in the genome longer, and hence we are more likely to find them in innocuous locations.

Both plant and animal hosts have evolved means for reducing the fitness impact of TEs, both by directly silencing them and by reducing their ability to transpose in the genome. It would appear that hosts in general are fairly tolerant of TEs in their genomes, since a sizable portion (30–80%) of the genome of many animals and plants is TEs.[60,61] When the host is able to stop their movement, TEs can simply be frozen in place, and it then can take millions of years for them to mutate away. The fitness of a TE is a combination of its ability to expand in numbers within a genome, to evade host defences, but also to avoid eroding host fitness too drastically. The effect of TEs in the genome is not entirely selfish. Because their insertion into the genome can disrupt gene function, sometimes those disruptions can have positive fitness value for the host. Many adaptive changes in *Drosophila*[62] and dogs[63] for example, are associated with TE insertions.

### B chromosomes

**Fig 5:**
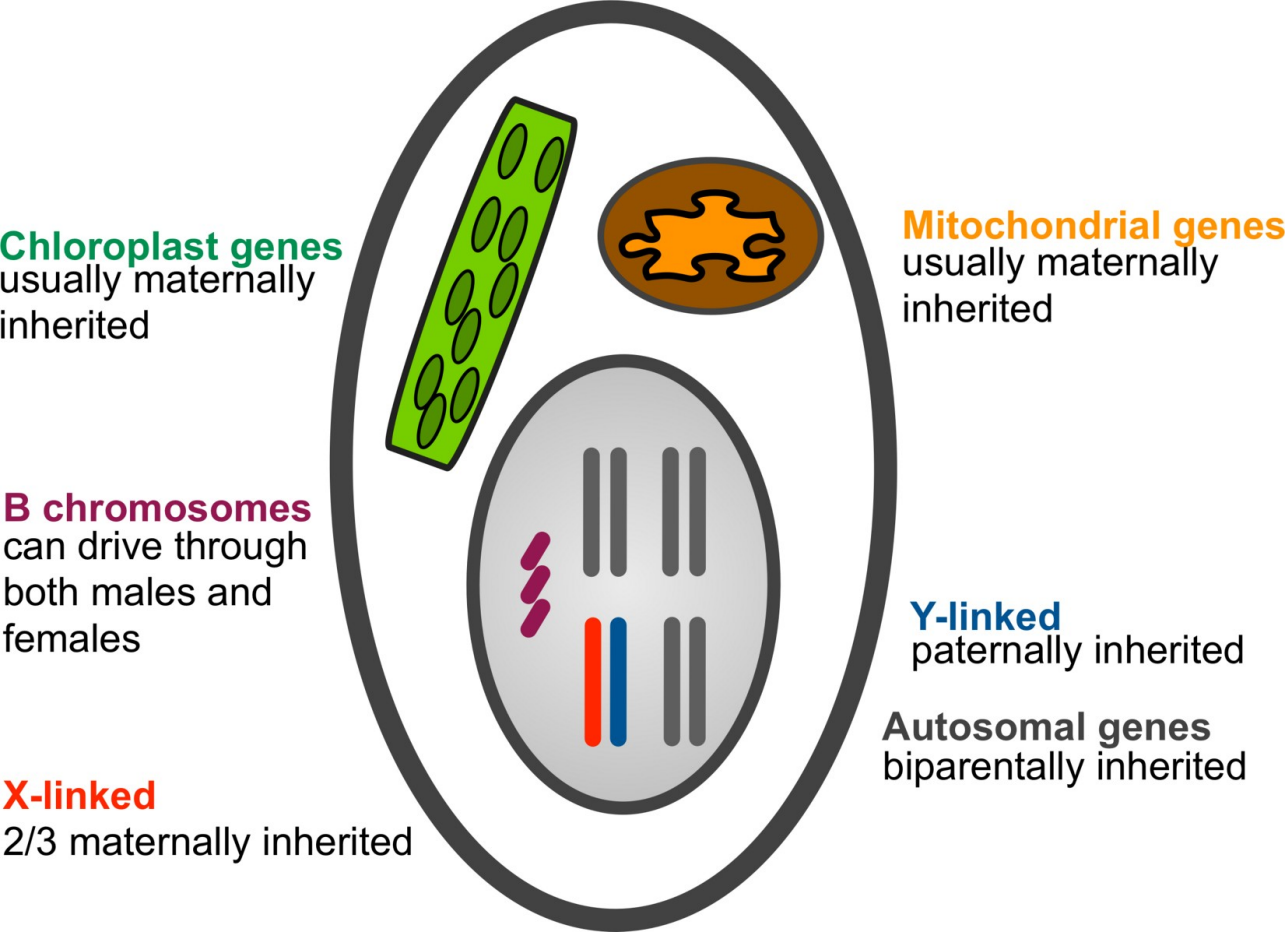
Genetic conflicts often arise because not all genes are inherited in the same way. Examples include cytoplasmic male sterility (see Selfish mitochondria). While mitochondrial and chloroplast genes are generally maternally inherited, B chromosomes can be preferentially transmitted through both males and females.

B chromosomes refer to chromosomes that are not required for the viability or fertility of the organism, but exist in addition to the normal (A) set.[64] They persist in the population and accumulate because they have the ability to propagate their own transmission independently of the A chromosomes (Fig 5). They often vary in copy number between individuals of the same species.

B chromosomes were first detected over a century ago.[65] Though typically smaller than normal chromosomes, their gene poor, heterochromatin-rich structure made them visible to early cytogenetic techniques. B chromosomes have been thoroughly studied and are estimated to occur in 15% of all eukaryotic species.[66] In general, they appear to be particularly common among eudicot plants, rare in mammals, and absent in birds.In 1945, they were the subject of Gunnar Östergren’s classic paper”Parasitic nature of extra fragment chromosomes”, where he argues that the variation in abundance of B chromosomes between and within species is because of the parasitic properties of the Bs (see above).[14] This was the first time genetic material was referred to as”parasitic” or”selfish”. B chromosome number correlates positively with genome size[67] and has also been linked to a decrease in egg production in the grasshopper *Eyprepocnemis plorans*.[68]

### Selfish mitochondria

Genomic conflicts often arise because not all genes are inherited in the same way. Probably the best example of this is the conflict between uniparentally (usually but not always, maternally) inherited mitochondrial and biparentally inherited nuclear genes. Indeed, one of the earliest clear statement about the possibility of genomic conflict was made by the English botanist Dan Lewis in reference to the conflict between maternally inherited mitochondrial and biparentally inherited nuclear genes over sex allocation in hermaphroditic plants (Fig 5).[16]

A single cell typically contains multiple mitochondria, creating a situation for competition over transmission. Uniparental inheritance been suggested to be a way to reduce the opportunity for selfish mitochondria to spread, as it ensures all mitochondria share the same genome, thus removing the opportunity for competition. [28,69,70] This view remains widely held, but has been challenged.[71] Why inheritance ended up being maternal, rather than paternal, is also much debated, but one key hypothesis is that the mutation rate is lower in female compared to male gametes.[72]

The conflict between mitochondrial and nuclear genes is especially easy to study in flowering plants.[73,74] Flowering plants are typically hermaphrodites,[75] and the conflict thus occurs within a single individual. Mitochondrial genes are typically only transmitted through female gametes, and therefore from their point of view the production of pollen leads to an evolutionary dead end. Any mitochondrial mutation that can affect the amount of resources the plant invests in the female reproductive functions at the expense of the male reproductive functions improves its own chance of transmission. Cytoplasmic male sterility is the loss of male fertility, typically through loss of functional pollen production, resulting from a mitochondrial mutation.[76] In many species where cytoplasmic male sterility occurs, the nuclear genome has evolved so-called restorer genes, which repress the effects of the cytoplasmic male sterility genes and restore the male function, making the plant a hermaphrodite again.[77,78]

The co-evolutionary arms race between selfish mitochondrial genes and nuclear compensatory alleles can often be detected by crossing individuals from different species that have different combinations of male sterility genes and nuclear restorers, resulting in hybrids with a mismatch.[79]

Another consequence of the maternal inheritance of the mitochondrial genome is the so-called Mother's Curse.[80] Because genes in the mitochondrial genome are strictly maternally inherited, mutations that are beneficial in females can spread in a population even if they are deleterious in males.[81] Explicit screens in fruit flies have successfully identified such female-neutral but male-harming mtDNA mutations.[82,83] Furthermore, a 2017 paper showed how a mitochondrial mutation causing Leber's hereditary optic neuropathy, a male-biased eye disease, was brought over by one of the *Filles du roi* that arrived in Quebec, Canada, in the 17th century and subsequently spread among many descendants.[84]

### Genomic imprinting

Another sort of conflict that genomes face is that between the mother and father competing for control of gene expression in the offspring, including the complete silencing of one parental allele. Due to differences in methylation status of gametes, there is an inherent asymmetry to the maternal and paternal genomes that can be used to drive a differential parent-of-origin expression. This results in a violation of Mendel’s rules at the level of expression, not transmission, but if the gene expression affects fitness, it can amount to a similar end result.

**Fig 6:**
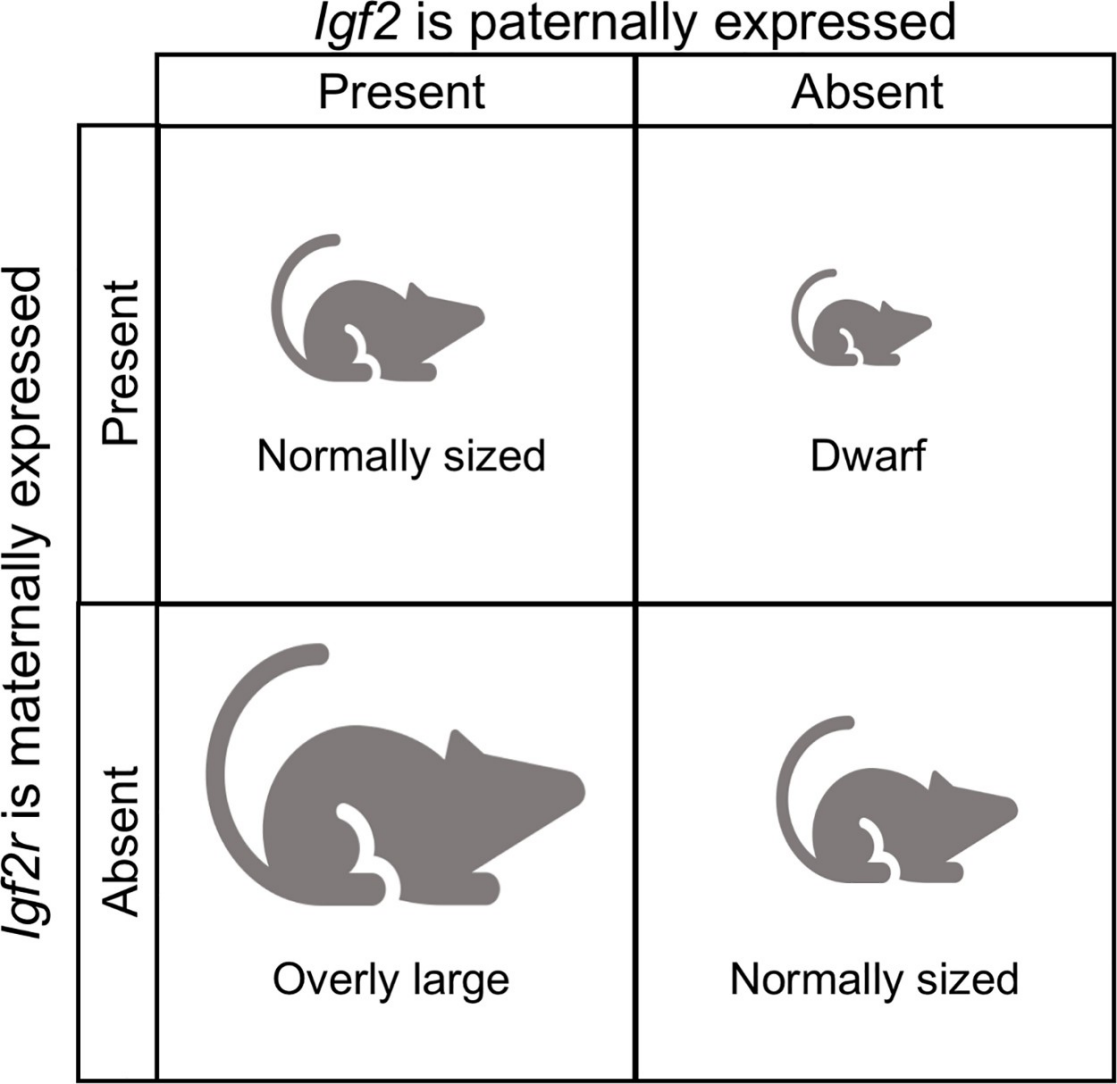
*Igf2* is an example of genomic imprinting. In mice, the insulin-like growth factor 2 gene, *Igf2*, which is linked to hormone production and increased offspring growth is paternally expressed (maternally silenced) and the insulin-like growth factor 2 receptor gene *Igf2r*, which binds the growth protein and so slows growth, is maternally expressed (paternally silenced). The offspring is normal sized when both genes are present, or both genes are absent. When the maternally expressed gene (*Igf2r*) is experimentally knocked out the offspring has an unusually large size, and when the paternally expressed gene (*Igf2*) is knocked out, the offspring is unusually small.

Imprinting seems like a maladaptive phenomenon, since it essentially means giving up diploidy, and heterozygotes for one defective allele are in trouble if the active allele is the one that is silenced. Several human diseases, such as Prader-Willi and Angelman syndromes, are associated with defects in imprinted genes. The asymmetry of maternal and paternal expression suggests that some kind of conflict between these two genomes might be driving the evolution of imprinting. In particular, several genes in placental mammals display expression of paternal genes that maximize offspring growth, and maternal genes that tend to keep that growth in check (Fig 6). Many other conflict-based theories about the evolution of genomic imprinting have been put forward.[85,86]

At the same time, genomic or sexual conflict are not the only possible mechanisms whereby imprinting can evolve.[87] Several molecular mechanisms for genomic imprinting have been described, and all have the aspect that maternally and paternally derived alleles are made to have distinct epigenetic marks, in particular the degree of methylation of cytosines. An important point to note regarding genomic imprinting is that it is quite heterogeneous, with different mechanisms and different consequences of having single parent-of-origin expression. For example, examining the imprinting status of closely related species allows one to see that a gene that is moved by an inversion into close proximity of imprinted genes may itself acquire an imprinted status, even if there is no particular fitness consequence of the imprinting.

### Greenbeards

A greenbeard gene is a gene that have the ability to recognize copies of itself in another individuals and then make its carrier act preferentially toward such individuals. The name itself comes from thought-experiment first presented by Bill Hamilton[88] and then it was developed and given its current name by Richard Dawkins in *The Selfish Gene*. The point of the thought experiment was to highlight that from a gene’s-eye view, it is not the genome-wide relatedness that matters (which is usually how kin selection operates, i.e. cooperative behavior is directed towards relatives), but the relatedness at the particular locus that underlies the social behavior.

**Fig 7:**
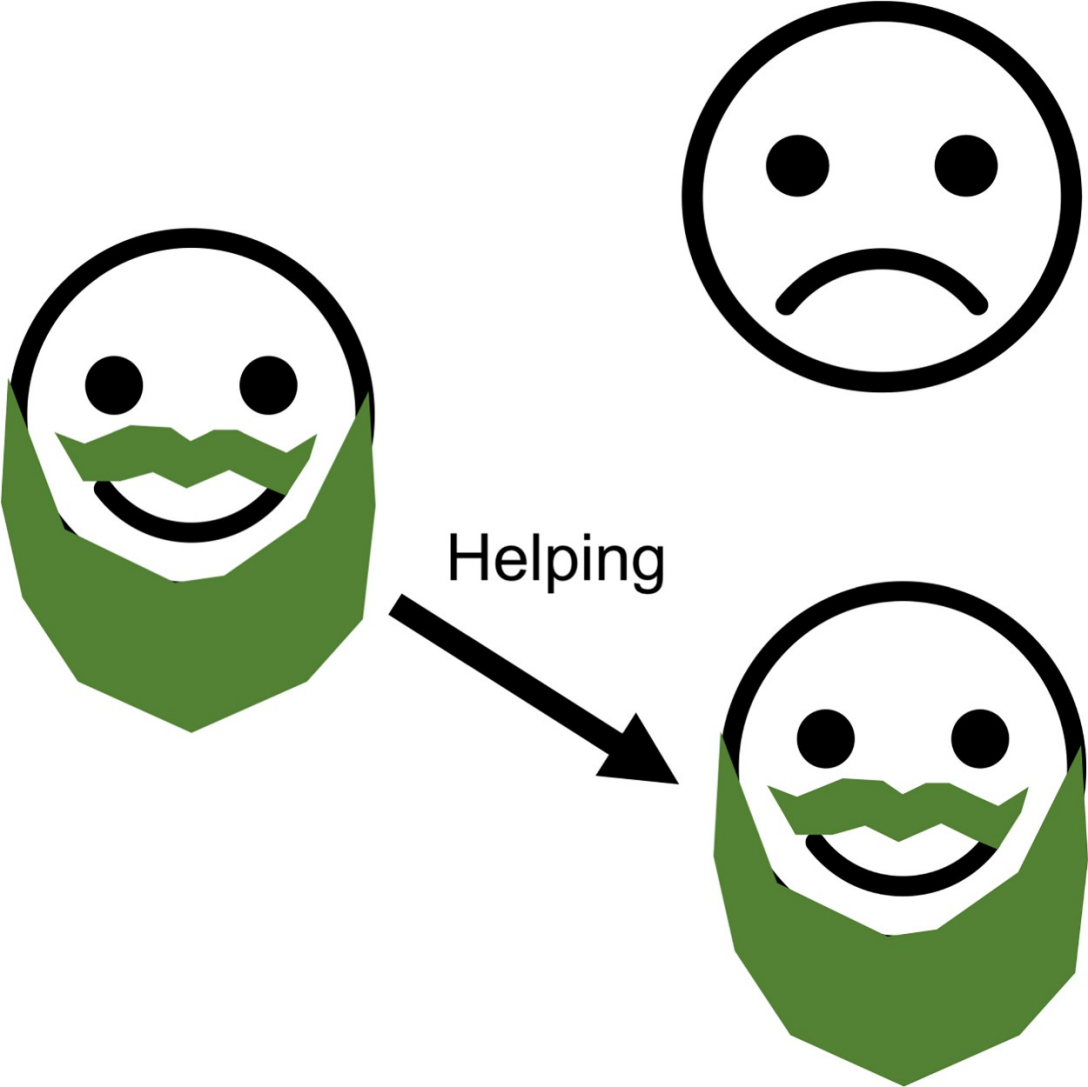
The simplest form of greenbeard mechanism. An individual with the greenbeard allele preferentially helps a fellow greenbeard individual.

Following Dawkins, a greenbeard is usually defined as a gene, or set of closely linked genes, that has three effects[89,90]:

1. It gives carriers of the gene a phenotypic label, such as a green beard.
2. The carrier is able to recognize other individuals with the same label.
3. The carrier then behaves altruistically towards individuals with the same label (Fig 7).

Greenbeards where long thought to be a fun theoretical idea with limited possibility of actually existing in nature. However, since its conception several examples have been identified, including in yeast[91], slime moulds[92], and fire ants[93].

There has been some debate whether greenbeard genes should be considered selfish genetic elements. [94–96] Conflict between a greenbeard locus and the rest of the genome can arise because during a given social interaction between two individuals, the relatedness at the greenbeard locus can be higher than at other loci in the genome. As a consequence, it may in the interest of the greenbeard locus to perform a costly social act, but not in the interest of the rest of the genome.

## Consequences to the host of selfish genetic elements

### Species extinction

Perhaps one of the clearest ways to see that the process of natural selection does not always have organismal fitness as the sole driver is when selfish genetic elements have their way without restriction. In such cases, selfish elements can, in principle, result in species extinction. This possibility was pointed out already in 1928 by Sergey Gershenson[13] and then in 1967, Bill Hamilton[97] developed a formal population genetic model for a case of segregation distortion of sex chromosomes driving a population to extinction. In particular, if a selfish element should be able to direct the production of sperm, such that males bearing the element on the Y chromosome would produce an excess of Y-bearing sperm, then in the absence of any countervailing force, this would ultimately result in the Y chromosome going to fixation in the population, producing an extremely male-biased sex ratio. In ecologically challenged species, such biased sex ratios imply that the conversion of resources to offspring becomes very inefficient, to the point of risking extinction.

### Speciation

Selfish genetic elements have been shown to play a role in speciation.[41,42,98] This could happen because the presence of selfish genetic elements can result in changes in morphology and/or life history, but ways by which the co-evolution between selfish genetic elements and their suppressors can cause reproductive isolation through so-called Bateson-Dobzhansky-Muller incompatibilities has received particular attention.

An early striking example of hybrid dysgenesis induced by a selfish genetic element was the *P* element in *Drosophila*.[99,100] If males carrying the *P* element were crossed to females lacking it, the resulting offspring suffered from reduced fitness. However, offspring of the reciprocal cross were normal, as would be expected since piRNAs are maternally inherited. The *P* element is typically present only in wild strains, and not in lab strains of *D*. *melanogaster*, as the latter were collected before the *P* elements were introduced into the species, probably from a closely related *Drosophila* species. The *P* element story is also a good example of how the rapid co-evolution between selfish genetic elements and their silencers can lead to incompatibilities on short evolutionary time scales, as little as within a few decades.

Several other examples of selfish genetic elements causing reproductive isolation have since been demonstrated. Crossing different species of *Arabidopsis* results in both higher activity of transposable elements[101] and disruption in imprinting,[102] both of which have been linked to fitness reduction in the resulting hybrids. Hybrid dysgenesis has also been shown to be caused by centromeric drive in barley[103] and in several species of angiosperms by mito-nuclear conflict.[104]

### Genome size variation

Attempts to understand the extraordinary variation in genome size (C-value)—animals vary 7,000 fold and land plants some 2,400-fold—has a long history in biology.[105] However, this variation is poorly correlated with gene number or any measure of organismal complexity, which led CA Thomas to coin the term C-value paradox in 1971.[106] The discovery of non-coding DNA resolved some of the paradox, and most current researchers now use the term”C-value enigma”.[107]

Two kinds of selfish genetic elements in particular have been shown to contribute to genome size variation: B chromosomes and transposable elements.[67,108] The contribution of transposable elements to the genome is especially well studied in plants.[60,61,109] A striking example is how the genome of the model organism *Arabidopsis thaliana* contains the same number of genes as that of the Norwegian spruce (*Picea abies*), around 30,000, but accumulation of transposons means that the genome of the latter is some 100 times larger. Transposable element abundance has also been to shown to cause the unusually large genomes found in salamanders.[110]

The presence of an abundance of transposable elements in many eukaryotic genomes was a central theme of the original selfish DNA papers mentioned above (See Conceptual developments). Most people quickly accepted the central message of those papers, that the existence of transposable elements can be explained by selfish selection at the gene level and there is no need to invoke individual level selection. However, the idea that organisms keep transposable elements around as genetic reservoir to “speed up evolution” or for other regulatory functions persists in some quarters.[111] In 2012, when the ENCODE Project published a paper claiming that 80% of the human genome can be assigned a function, a claim interpreted by many as the death of the idea of junk DNA, this debate was reignited.[112,113]

## Applications of selfish genetic elements in agriculture and biotechnology

### Cytoplasmic male sterility in plant breeding

A common problem for plant breeders is unwanted self-fertilization. This is particularly a problem when breeders try to cross two different strains to create a new hybrid strain. One way to avoid this is manual emasculation, i.e. physically removing anthers to render the individual male sterile. Cytoplasmic male sterility offers an alternative to this laborious exercise.[114]Breeders cross a strain that carries a cytoplasmic male sterility mutation with a strain that does not, the latter acting as the pollen donor. If the hybrid offspring are to be harvested for their seed (like maize), and therefore needs to be male fertile, the parental strains need to be homozygous for the restorer allele. In contrast, in species that harvested for their vegetable parts, like onions, this is not an issue. This technique has been used in a wide variety of crops, including rice, maize, sunflower, wheat, and cotton.[115]

### PiggyBac vectors

While many transposable elements seem to do no good for the host, some transposable elements have been “tamed” by molecular biologists so that the elements can be made to insert and excise at the will of the scientist. Such elements are especially useful for doing genetic manipulations, like inserting foreign DNA into the genomes of a variety of organisms.

One excellent example of this is PiggyBac, a transposable element that can efficiently move between cloning vectors and chromosomes using a "cut and paste" mechanism.[116] The investigator constructs a PiggyBac element with the desired payload spliced in, and a second element (the PiggyBac transposase), located on another plasmid vector, can be co-transfected into the target cell. The PiggyBac transposase cuts at the inverted terminal repeat sequences located on both ends of the PiggyBac vector and efficiently moves the contents from the original sites and integrates them into chromosomal positions where the sequence TTAA is found. The three things that make PiggyBac so useful are the remarkably high efficiency of this cut-and-paste operation, its ability to take payloads up to 200 kb in size, and its ability to leave a perfectly seamless cut from a genomic site, leaving no sequences or mutations behind.[117]

### CRISPR gene drive and homing endonuclease systems

CRISPR allows the construction of artificial homing endonucleases, where the construct produces guide RNAs that cut the target gene, and homologous flanking sequences then allow insertion of the same construct harboring the Cas9 gene and the guide RNAs. Such gene drives ought to have the ability to rapidly spread in a population (see section below on the theory of gene drives), and one practical application of such a system that has been proposed is to apply it to a pest population, greatly reducing its numbers or even driving it extinct.[56]This has not yet been attempted in the field, but gene drive constructs have been tested in the lab, and the ability to insert into the wild-type homologous allele in heterozygotes for the gene drive has been demonstrated.[55] Unfortunately, the double-strand break that is introduced by Cas9 can be corrected by Homology directed repair, which would make a perfect copy of the drive, or by Non-homologous end joining, which would produce “resistant” alleles unable to further propagate themselves. When Cas9 is expressed outside of meiosis, it seems like non-homologous end joining predominates, making this the biggest hurdle to practical application of gene drives.[118]

## Mathematical theory of selfish genetic elements

Much of the confusion regarding ideas about selfish genetic elements center on the use of language and the way the elements and their evolutionary dynamics are described.[119] The beauty of mathematical models is that we can all agree on the assumptions and the rules for establishing mathematical statements about the expected dynamics of the elements in populations, and then explore the consequences of having such elements in genomes. The mathematics can define very crisply the different classes of elements by their precise behavior within a population, sidestepping any distracting verbiage about the inner hopes and desires of greedy selfish genes. There are many good examples of this approach, and we will focus on segregation distorters, gene drive systems and transposable elements.

### Segregation distorters

The mouse t-allele is a classic example of a segregation distorter system that has been modeled in great detail.[51,120] Heterozygotes for a t-haplotype produce >90% of their gametes bearing the t (segregation distortion, see above), and homozygotes for a t-haplotype die as embryos. This can result in a stable polymorphism, with an equilibrium frequency that depends on the drive strength and direct fitness impacts of t-haplotypes. This is a common theme in the mathematics of segregation distorters: virtually every example we know entails a countervailing selective effect, without which the allele with biased transmission would go to fixation and the segregation distortion would no longer be manifested. Whenever sex chromosomes undergo segregation distortion, the population sex ratio is altered, making these systems particularly interesting. Two classic examples of segregation distortion involving sex chromosomes include the “Sex Ratio” X chromosomes of *Drosophila pseudoobscura*[49] and Y chromosome drive suppressors of *Drosophila mediopunctata*.[121] A crucial point about the theory of segregation distorters is that just because there are fitness effects acting against the distorter, this does not guarantee that there will be a stable polymorphism. In fact, some sex chromosome drivers can produce frequency dynamics with wild oscillations and cycles.[122]

### Gene drive systems

The idea of spreading a gene into a population as a means of population control is actually quite old, and models for the dynamics of introduced compound chromosomes date back to the 1970s.[123] More recently, the population genetics theory for homing endonucleases and CRISPR-based gene drives has become much more advanced.[52,124] An important component of modeling these processes in natural populations is to consider the genetic response in the target population. For one thing, any natural population will harbor standing genetic variation, and that variation might well include polymorphism in the sequences homologous to the guide RNAs, or the homology arms that are meant to direct the repair. In addition, different hosts and different constructs may have quite different rates of non-homologous end joining, the form of repair that results in broken or resistant alleles that no longer spread. Full accomodation of the host factors presents considerable challenge for getting a gene drive construct to go to fixation, and Unckless and colleagues[125] show that in fact the current constructs are quite far from being able to attain even moderate frequencies in natural populations. This is another excellent example showing that just because an element appears to have a strong selfish transmission advantage, whether it can successfully spread may depend on subtle configurations of other parameters in the population.

### Transposable elements

To model the dynamics of transposable elements (TEs) within a genome, one has to realize that the elements behave like a population within each genome, and they can jump from one haploid genome to another by horizontal transfer. The mathematics has to describe the rates and dependencies of these transfer events. It was observed early on that the rate of jumping of many TEs varies with copy number, and so the first models simply used an empirical function for the rate of transposition. This had the advantage that it could be measured by experiments in the lab, but it left open the question of why the rate differs among elements and differs with copy number. Stan Sawyer and Daniel L. Hartl[126] fitted models of this sort to a variety of bacterial TEs, and obtained quite good fits between copy number and transmission rate and the population-wide incidence of the TEs. TEs in higher organisms, like *Drosophila*, have a very different dynamics because of sex, and Brian Charlesworth, Deborah Charlesworth, Charles Langley, John Brookfield and others[34,127,128] modeled TE copy number evolution in *Drosophila* and other species. What is impressive about all these modeling efforts is how well they fitted empirical data, given that this was decades before discovery of the fact that the host fly has a powerful defense mechanism in the form of piRNAs. Incorporation of host defense along with TE dynamics into evolutionary models of TE regulation is still in its infancy.[129]

## Future directions

Selfish genetic elements have gone from being seen as genetic oddities with little significance to be considered major players in evolution. We now know that their presence and co-evolution with suppressors can affect a range of features at the genome, phenotype, and population levels. While the influx of whole genome data has done much to bring this shift about, particularly for the study of transposable elements, developments in mathematical modelling has also played a key role. The adaption of CRISPR technologies to engineer gene drive systems for population control has added a new rapidly advancing applied dimension to the study of selfish genetic elements.

## Supporting information

S1 Text Version history of the text file. (XML)

S2 Text Peer reviews and response to reviews. (XML)
